# Association of high microvessel α_v_β_3_ and low PTEN with poor outcome in stage 3 neuroblastoma: rationale for using first in class dual PI3K/BRD4 inhibitor, SF1126

**DOI:** 10.18632/oncotarget.13386

**Published:** 2016-11-18

**Authors:** Anat Erdreich-Epstein, Alok R. Singh, Shweta Joshi, Francisco M. Vega, Pinzheng Guo, Jingying Xu, Susan Groshen, Wei Ye, Melissa Millard, Mihaela Campan, Guillermo Morales, Joseph R. Garlich, Peter W. Laird, Robert C. Seeger, Hiroyuki Shimada, Donald L. Durden

**Affiliations:** ^1^ Department of Pediatrics, Children's Hospital Los Angeles and University of Southern California Keck School of Medicine, Los Angeles, California, USA; ^2^ Department of Pathology, Children's Hospital Los Angeles and University of Southern California Keck School of Medicine, Los Angeles, California, USA; ^3^ Department of Pediatrics, Moores Cancer Center, University of California San Diego, California, USA; ^4^ Instituto de Biomedicina de Sevilla, IBiS/HUVR/CSIC/Universidad de Sevilla and Department of Medical Physiology and Biophysics, Universidad de Sevilla, Spain; ^5^ Department of Preventive Medicine, Keck School of Medicine, Los Angeles, California, USA; ^6^ Department of Surgery University of Southern California, Keck School of Medicine, Los Angeles, California, USA; ^7^ SignalRx Pharmaceuticals, San Diego, California, USA; ^8^ USC Epigenome Center, University of Southern California, Keck School of Medicine, Los Angeles, California, USA; ^9^ Department of Pediatrics, UCSD School of Medicine and Rady Children's Hospital San Diego, California, USA; ^10^ Current Address: Van Andel Research Institute, Grand Rapids, Michigan, USA

**Keywords:** angiogenesis, integrin α_v_β_3_, neuroblastoma, PI3-kinase inhibitors, BRD4

## Abstract

Neuroblastoma (NB) is the most common extracranial solid tumor in children. Our previous studies showed that the angiogenic integrin α_v_β_3_ was increased in high-risk metastatic (stage 4) NB compared with localized neuroblastomas. Herein, we show that integrin α_v_β_3_ was expressed on 68% of microvessels in MYCN-amplified stage 3 neuroblastomas, but only on 34% (means) in MYCN-non-amplified tumors (*p* < 0.001; *n* = 54). PTEN, a tumor suppressor involved in α_v_β_3_ signaling, was expressed in neuroblastomas either diffusely, focally or not at all (immunohistochemistry). Integrin α_v_β_3_ was expressed on 60% of tumor microvessels when PTEN was negative or focal, as compared to 32% of microvessels in tumors with diffuse PTEN expression (*p* < 0.001). In a MYCN transgenic mouse model, loss of one allele of PTEN promoted tumor growth, illustrating the potential role of PTEN in neuroblastoma pathogenesis. Interestingly, we report the novel dual PI-3K/BRD4 activity of SF1126 (originally developed as an RGD-conjugated pan PI3K inhibitor). SF1126 inhibits BRD4 bromodomain binding to acetylated lysine residues with histone H3 as well as PI3K activity in the MYCN amplified neuroblastoma cell line IMR-32. Moreover, SF1126 suppressed MYCN expression and MYCN associated transcriptional activity in IMR-32 and CHLA136, resulting in overall decrease in neuroblastoma cell viability. Finally, treatment of neuroblastoma tumors with SF1126 inhibited neuroblastoma growth *in vivo*. These data suggest integrin α_v_β_3_, MYCN/BRD4 and PTEN/PI3K/AKT signaling as biomarkers and hence therapeutic targets in neuroblastoma and support testing of the RGD integrin α_v_β_3_-targeted PI-3K/BRD4 inhibitor, SF1126 as a therapeutic strategy in this specific subgroup of high risk neuroblastoma.

## INTRODUCTION

Neuroblastoma (NB) is the most common and deadly extracranial solid tumor in children, arising from the sympathetic nervous system and accounting for 8–10% of all childhood cancers and 15% of deaths from pediatric tumors [1]. Patients with high-risk neuroblastoma have poor outcome despite intensive treatment [2]. In many cancers, including neuroblastoma, increased angiogenesis is associated with more aggressive tumors and poorer prognosis [3, 4].

Integrins are a family of non-covalently linked α- and β-heterodimeric cell surface adhesion receptors that regulate critical cellular functions such as migration, cell growth, differentiation and survival. Integrin α_v_β_3_ is preferentially expressed on angiogenic blood vessels in some cancers, where its expression is associated with tumor aggressiveness and worse prognosis [5]. Inhibition of vascular integrins α_v_β_3_ and αvβ5 results in apoptosis of angiogenic endothelial cells, inhibition of tumor angiogenesis, and impaired tumor growth [6–8], potentially supporting clinical use of integrin-based therapy. However, inhibitors of integrin α_v_β_3_ have not shown much promise in clinical trials [9, 10] suggesting that a different approach is needed to therapeutically exploit the angiogenic expression of integrin α_v_β_3_ in cancer.

Neuroblastoma response to therapy varies depending on clinical stage and tumor biology. Prior to the advent of high-throughput large scale molecular analyses and establishment of the International Neuroblastoma Risk Group (INRG) Task Force, neuroblastoma risk stratification varied between the groups studying it [11]. Risk stratification can help predict prognosis and tailor therapy for patients and continues to evolve as molecular understanding of neuroblastoma biology increases [11–13]. For CCG/COG (Children's Cancer Group/Children's Oncology Group) neuroblastoma studies prior to the INRG stratification, in addition to clinical stage, other factors, including age, amplification of the MYCN oncogene and tumor histology (Shimada classification), were used in assigning risk groups (high-risk, intermediate-risk, or low-risk) [14, 15]. Based on clinical experience, the majority of localized (stage 1 and 2) tumors were considered biologically favorable and in a low-risk group, and the majority of disseminated (stage 4) tumors were biologically unfavorable and in a high-risk group. Tumors included in the study presented here were stage 3 neuroblastomas, defined as large tumors crossing the midline and without distant metastases [14, 15].

We previously showed that integrin α_v_β_3_ expression in blood vessels in high-risk metastatic (stage 4) neuroblastomas was higher than in localized tumors (stages 1–2) [5]. A challenging group of patients are those with stage 3 neuroblastoma that comprises a mix of biologically- favorable and biologically-unfavorable tumors for which it has been complex to assign risk group and predict clinical outcome. Of the five stage 3 neuroblastomas in our prior series [5], microvessel integrin α_v_β_3_ expression was high in the three MYCN-amplified tumors (mean 87% of microvessels, 95% CI 79%–94%), but low in the two MYCN-non-amplified ones (mean 20% of microvessels). Here we expanded the analysis of integrin α_v_β_3_ microvascular expression to 54 stage 3 neuroblastomas in order to assess if integrin α_v_β_3_ could further stratify risk in this challenging group of stage 3 patients. Since the tumor suppressor gene and regulator of angiogenesis, PTEN [16] has an important role in control of endothelial integrin α_v_β_3_ function [17, 18], we also evaluated PTEN expression in this set of stage 3 neuroblastomas. We also determined the effect of manipulation of the PTEN/PI3K/AKT signaling pathway on growth of neuroblastoma xenografts *in vivo* and *in vitro* by treatment with an RGD-targeted dual PI3K/BRD4 inhibitor, with anti-tumor and anti-angiogenic activity, SF1126.

SF1126, a pan-PI-3K inhibitor, has shown anti-tumor and anti-angiogenic activity in a number of xenograft models [19–23]. Furthermore, this drug has recently been shown to be safe (no dose limiting toxicity or hepatotoxicity) and have considerable efficacy in B cell malignancies and a variety of solid tumors in a Phase I clinical trial [24]. SF1126 is an RGDS-conjugated LY294002 prodrug, which is designed to exhibit increased solubility and bind to specific integrins within the tumor compartment, resulting in enhanced delivery of the active compound to the tumor vasculature and tumor [22]. In a recent study LY294002, the active moiety of SF1126, was cocrystallized in the active site of BRD4 and inhibited BET bromodomain binding to acetylated lysine binding sites on histones within chromatin [25]. The bromodomain and extraterminal domain (BET) proteins recently emerged as important therapeutic targets in NUT midline carcinoma and several types of hematopoietic cancers [26–29]. Bromodomains are protein motifs that primarily bind to acetylated lysine residues, including those on histone tails [30]. Through this interaction, bromodomain-containing proteins direct the assembly of nuclear macromolecular complexes to specific sites on chromatin that regulate key biologic processes including DNA replication, DNA damage repair, chromatin remodeling, and transcription regulation [30, 31]. The BET family proteins (BRD2, BRD3, BRD4, BRDT) contain 2 amino-terminal bromodomains and have recently been recognized in the literature as a therapeutic strategy to target MYCN [29]. MYCN transcription factor is frequently up-regulated in a variety of human cancers [32], including neuroblastoma [33]. The pathologic activation of MYCN plays a central role in high-risk neuroblastoma, with *MYCN* amplification identified in 25% of primary neuroblastoma tumors and nearly half of high-risk cases [1, 34, 35]. Although bromodomain inhibitors have captured considerable attention for the treatment of MYC and MYCN dependent cancers, other laboratories have suggested that dual inhibition of BRD4 and PI-3K/AKT will maximally inhibit the MYC oncogene via effects on both MYCN transcription and protein degradation [36]. In this report, we confirm the dual inhibitory activity of SF1126 toward PI-3K and BRD4 in NB. The aim of this study was to evaluate the role of PTEN/PI-3K and the BRD4/MYCN signaling axis and a “first in class” dual PI-3K/BRD4 inhibitor, SF1126 as biomarkers and a therapeutic strategy, respectively for the treatment of MYCN dependent high risk neuroblastoma.

## RESULTS

### More microvessels in aggressive stage 3 neuroblastoma express integrin α_v_β_3_ compared to less aggressive stage 3 neuroblastoma

**Figure 1:**
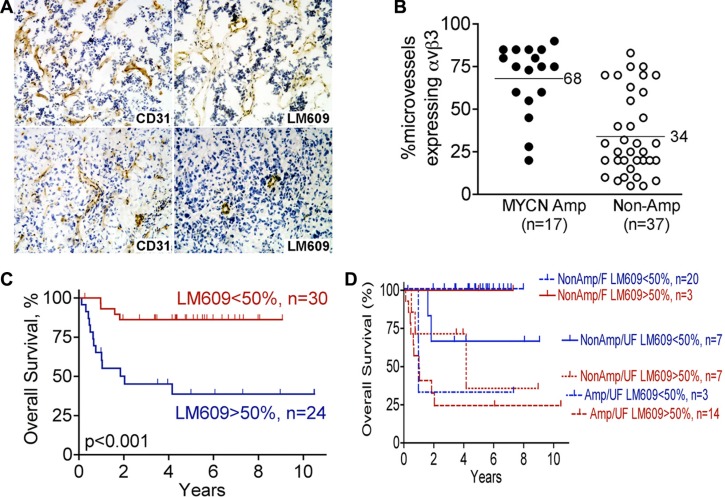
Higher expression of integrin α_v_β_3_ on microvessels of high-risk stage 3 neuroblastoma Serial frozen sections of 54 cases of stage 3 neuroblastoma, as summarized in Table 1, were stained for integrin α_v_β_3_ (mAb LM609) and CD31. The fraction of all vessels (anti-CD31) that also express integrin α_v_β_3_ (LM609 antibody) was determined as detailed in “Materials and Methods”, and used for the analysis in panels B–D. (**A**) Example of stage 3 neuroblastomas with high (top panels) or low (bottom panels) microvessel α_v_β_3_ expression: immunohistochemistry for CD31 is in left panels and α_v_β_3_ (LM609 antibody) in right panels. Photographed at 100× magnification. (**B**) Scatter plot of percent microvessels expressing integrin α_v_β_3_ as function of MYCN amplification status in the 54 stage 3 neuroblastomas analyzed as in panel A. ● – MYCN amplified tumors; ○ – MYCN non-amplified tumors. *p* < 0.001 by unpaired *t*-test. (**C**) Kaplan-Meier plot of overall survival of patients with stage 3 neuroblastoma according to percent microvessels expressing integrin α_v_β_3_ (< 50% or > 50% of vessels staining with LM609 antibody). *p* < 0.001. (**D**) Kaplan-Meier plot of overall survival comparing < 50% or > 50% microvessels expressing integrin α_v_β_3_ (LM609 antibody) in the three risk groups defined by MYCN-amplification and Shimada classification (MYCN amplified/unfavorable Shimada, MYCN non-amplified/unfavorable and Shimada MYCN non-amplified/favorable Shimada). *p* = 0.58 between < 50% and > 50% α_v_β_3_ expression after adjusting for the risk groups.

To determine frequency of integrin α_v_β_3_- expressing microvessels in stage 3 neuroblastoma, we examined 54 primary tumor specimens obtained at time of diagnosis. We examined contiguous sections by immunohistochemistry using anti-CD31 (PECAM-1) to detect all vessels, and LM609 antibody to detect integrin α_v_β_3_ and determine the proportion of CD31-positive microvessels that express α_v_β_3_ (Figure 1A). Notably, CD31 and integrin α_v_β_3_ were only expressed on blood vessels but not on the tumor cells themselves (Figure 1A). Table 1 provides a summary of the proportion of microvessels expressing integrin α_v_β_3_ as a percentage of all CD31-positive microvessels. The main finding in this analysis is that on average, integrin α_v_β_3_ was expressed on 68% (95% CI 57%–79%; *n* = 17) of microvessels in stage 3 MYCN-amplified (high risk) neuroblastomas, but only on 34% (95% CI 26%–42%, *n* = 34, *p* < 0.001) of microvessels in MYCN-non-amplified ones (Table 1; Figure 1B). Further subdividing the groups to compare MYCN-amplification as well as Shimada classification, expression of integrin α_v_β_3_ continued to be significantly higher in the more aggressive tumors as follows: In neuroblastomas with amplified MYCN and unfavorable Shimada classification, integrin α_v_β_3_ was expressed on 68% of all microvessels (95% CI 57%–79%, *n* = 17, treated with high risk protocols, with or without BMT); In tumors with non-amplified MYCN and unfavorable Shimada, integrin α_v_β_3_ was expressed on 44% of microvessels (95% CI 33%–56%, *n* = 14, of whom 13 patients were ≥ 12 month old at diagnosis and treated according to the high risk protocol with or without BMT, and one patient > 12 months old at diagnosis, considered to have intermediate risk tumor, and treated with conventional chemotherapy); In tumors with non-amplified MYCN and favorable Shimada, integrin α_v_β_3_ was expressed on only 28% of microvessels (95% CI 19%–37%, *n* = 23; intermediate risk tumors, treated with conventional chemotherapy). For each of the pair-wise comparisons among these groups *p <* 0.05 (Table 1).

**Table 1:** Expression of integrin α_v_β_3_ on tumor microvessels is associated with poor prognostic markers in patients with stage 3 neuroblastoma

	No. of patients (%)	Mean % microvessels expressingα_v_β_3_ (95% confidence interval)	*P*-value from *t*-test or ANOVA
MYCN			
Non-amplified	37 (69%)	34% (26% – 42%)	< 0.001
Amplified	17 (31%)	68% (57% – 79%)	
Shimada Classification			
Favorable	23 (43%)	28% (20% – 36%)	< 0.001
Unfavorable	31 (57%)	57% (48% – 67%)	
Age			
≤12 months	14 (26%)	35% (22% – 48%)	0.11
≥12 months	40 (74%)	48% (39% – 58%)	
≤18 months	23 (43%)	38% (27% – 49%)	0.11
≥18 months	31 (57%)	50% (40% – 60%)	
MYCN and Shimada classification			
Non-amp/favorable (intermediate risk)	23 (43%)	28% (19% – 37%)	< 0.001*
Non-amp/unfavorable (all but one are ≥ 12 month old)	14 (26%)	44% (33% – 56%)	
Amp/favorable	0		
Amp/unfavorable (high risk)	17 (31%)	68% (57% – 79%)	
PTEN expression			
Diffuse	28 (53%)	32% (23% – 42%)	< 0.001
Focal or negative	25 (47%)	60% (51% – 69%)	

*P-value for non-amp/favorable vs. non-amp/unfavorable = 0.033.

*P*-value for non-amp/unfavorable vs. amp/unfavorable = 0.005.

*P*-value for non-amp/favorable vs. amp/unfavorable < 0.001.

Higher percentage of microvessels expressing integrin αvβ3 was significantly associated with higher risk of fatal outcome univariately (*p* < 0.001 for overall survival; Figure 1C). However, after adjusting for MYCN and Shimada classification, microvessel expression of integrin α_v_β_3_ did not provide additional prognostic information for overall survival (*p* = 0.58 from the log-rank test stratified by MYCN-amplified/unfavorable Shimada, MYCN-non-amplified/unfavorable Shimada and MYCN-non-amplified/favorable Shimada; Figure 1D). This suggests that microvessel expression of integrin α_v_β_3_, an indicator of active angiogenesis, may be biologically linked to MYCN and Shimada classification in conferring higher risk biology to these tumors.

### PTEN is diffusely expressed in less aggressive stage 3 neuroblastoma, but only focally- expressed, or not expressed at all, in the more aggressive stage 3 ones

**Figure 2:**
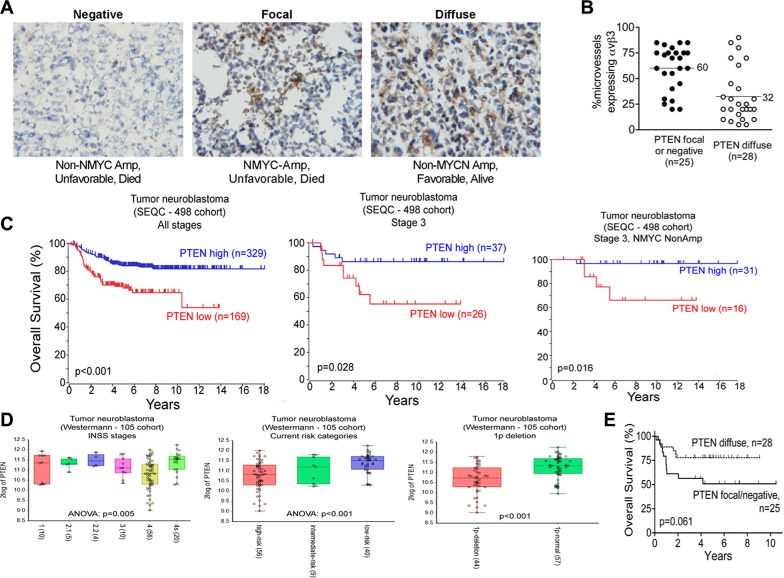
The tumor suppressor gene, PTEN is expressed in stage 3 neuroblastoma tumors Frozen sections of 53 cases of stage 3 neuroblastoma, contiguous to those analyzed in Figure 1, were stained for PTEN as detailed in “Materials and Methods” and used for the analysis in panels (**A**–**C**). (A) Examples of the three immunohistochemical staining patterns of PTEN in the stage 3 neuroblastomas. Left panel: negatively-staining tumor; Middle panel: focally-positive tumor; Right panel: diffusely positive tumor. Images were photographed at 400× magnification. (B) Kaplan-Meier plot for overall survival as function of PTEN staining pattern. *p* = 0.061 by the log-rank test. (C) Scatter plot of percent microvessels expressing integrin α_v_β_3_ as a function of PTEN staining pattern. ● – Focal or negative PTEN stain; ○ – Diffusely positive PTEN stain. *p* < 0.001 by unpaired *t*-test. Middle panel: focally-positive tumor; Right panel: diffusely positive tumor. Images were photographed at 400× magnification. (B) Kaplan-Meier plot for overall survival grouped by PTEN staining pattern. *p* = 0.061 by log-rank test. (C) Kaplan-Meier plots for overall survival as function of PTEN expression in a cohort of neuroblastoma patient samples (498 samples) or a subgroup of it, as indicated. (**D**) Plots showing PTEN expression on different subset of neuroblastoma patient samples from the Westermann cohort. Patients grouped by INSS stages, current risk stratification stages or the presence of chromosome 1p deletion are shown. *P* value for ANOVA or *T*-test is shown in each plot. (**E**) Scatter plot of percent microvessels expressing integrin α_v_β_3_ as a function of PTEN staining pattern. ● – Focal or negative PTEN stain; ○ – Diffusely positive PTEN stain. *p* < 0.001 by unpaired *t*-test. Middle panel: focally-positive tumor; Right panel: diffusely positive tumor. Images were photographed at 400× magnification. B. Kaplan-Meier plot for overall survival grouped by PTEN staining pattern. *p* = 0.061 by log-rank test. C. Scatter plot of percent microvessels expressing integrin α_v_β_3_ grouped by PTEN staining pattern. ● ● – Focal or negative PTEN stain; ○○ – Diffusely positive PTEN stain. *p* < 0.0001 by unpaired *t*-test.

Tumor angiogenesis is regulated by multiple factors, including integrin α_v_β_3_. The PI3K/AKT pathway is also critical in angiogenesis, with both PTEN and integrin α_v_β_3_ regulating angiogenic signaling interdependently in the PI3K/AKT pathway [16, 17, 37]. Importantly, the tumor suppressor PTEN, a key regulator of the PI3K/AKT cell survival pathway, is deleted in many tumor types [38]. We therefore examined the expression of PTEN by immunohistochemistry using frozen sections contiguous to the sections we analyzed in Table 1 and Figure 1 (53 of the 54 tumors were available). PTEN was expressed by the neuroblastoma cells themselves in three distinct patterns: diffusely in the whole tumor, focally by small groups of tumor cells in different areas of the tumor, or in a minority of the tumors, not expressed at all (Figure 2A; Table 2). Interestingly, 83% (19 of 23) of the MYCN-non-amplified/favorable histology neuroblastomas showed diffuse PTEN expression, whereas only 18% (3 of 17) of the MYCN-amplified/unfavorable histology tumors showed this diffuse expression of PTEN. Conversely, only 17% (4 of 23) of the MYCN- non-amplified/favorable histology neuroblastomas showed focal or negative expression of PTEN, whereas 82% (14 of 17) of the ones with MYCN-amplified/unfavorable histology had this limited PTEN expression (Table 2). Patients with MYCN-non-amplified/unfavorable histology neuroblastomas, who would be expected to have prognosis intermediate between these two groups, showed similar numbers of tumors with diffuse PTEN staining (*n* = 6, 46%) as with focal or negative PTEN staining (*n* = 7, 54%). Examination of overall survival in this group of stage 3 neuroblastoma patients univariately showed a trend, but not a significant difference (*p* = 0.061), toward better survival in patients whose neuroblastomas displayed diffuse PTEN expression compared to focal or negative expression of this tumor suppressor gene (Figure 2B). In other set of experiments, we used R2: Genomics Analysis and Visualization Platform (http://r2.amc.nl; Academic Medical Centre, Amsterdam), where we analyzed the overall survival of patients according to PTEN expression in an expression dataset obtained from a cohort of 498 neuroblastoma patient samples (Figure 2C). Interestingly, PTEN low expression significantly stratify patients with lower survival in the complete cohort or in stage 3 neuroblastoma patients (*p* < 0.001 and *p* = 0.028 respectively. Interestingly, PTEN expression also stratify patients according to their survival when only the stage 3, MYCN non-amplified patients were considered (*p* = 0.016), indicating that low expression of PTEN could be a marker for stage 3 patients with worse outcome, independently of MYCN amplification. Low PTEN expression was also found significantly correlating with high grades on another cohort of neuroblastoma patients classified under the current risk stratification categories (Figure 2D).

**Table 2:** Lower risk features are associated with a diffuse pattern of expression of PTEN in patients with stage 3 neuroblastoma

	No.of patients (% of 53)	# of tumors with PTEN pattern(%, across*)		*P*-value, Chi-square test
		Diffuse	Focal or negative	
Total	53 (100%)	28 (53%)	25 (47%)	
MYCN				
Non-amplified	36 (68%)	25 (69%)	11 (31%)	< 0.001
Amplified	17 (32%)	3 (18%)	14 (82%)	
Shimada Classification				
Favorable	23 (43%)	19 (83%)	4 (17%)	< 0.001
Unfavorable	30 (57%)	9 (30%)	21 (70%)	
Age				
≤12 months	14 (26%)	9 (64%)	5 (36%)	0.28
≥12 months	39 (74%)	19 (49%)	20 (51%)	
≤18 months	22 (42%)	14 (64%)	8 (36%)	0.25
≥18 months	31 (58%)	14 (45%)	17 (55%)	
MYCN and Shimada classification				
Non-amp/favorable (intermediate risk)	23 (43%)	19 (83%)	4 (17%)	< 0.001
Non-amp/unfavorable (all but one are ≥ 12 month old)	13 (25%)	6 (46%)	7 (54%)	
Amp/favorable	0			
Amp/unfavorable (high risk)	17 (32%)	3 (18%)	14 (82%)	

*Percentages in the “No. of patients” column refers to percentage out of total 53 patients. Percentages in the PTEN expression columns refers to the percent of patients with that pattern of PTEN staining in that specific risk category (i.e., across the lines).

To examine if the variation in PTEN expression found by immunohistochemistry may be due to methylation of the PTEN promoter in some of the tumor cells, we analyzed tumor DNA for methylation in the PTEN promoter, in comparison with two genes known to be methylated in neuroblastoma (RASSF1A, MTHFR [39, 40]. Tissue from 19 of the 53 stage 3 neuroblastomas was available for analysis, and included 9 tumors that by immunohistochemistry showed focal or negative expression of PTEN and 10 that showed diffuse expression of PTEN. This panel was also representative of the whole group in that it was comprised of stage 3 neuroblastomas with MYCN amplification or without it, with unfavorable or favorable Shimada histology, and tumors from patients who survived their tumors or succumbed to them. While the genes used as positive controls showed degrees of DNA methylation as expected, we found no methylation of the PTEN promoter in any of the 19 tumors analyzed in this panel (unpublished data). Thus, in this panel of stage 3 neuroblastoma tumors, PTEN expression was not regulated by DNA methylation of its promoter.

In view of the reported co-signaling between integrin α_v_β_3_ and PTEN and the expression of integrin α_v_β_3_ on neuroblastoma capillaries and PTEN in the tumor cells, we next examined if there was association between expression of integrin α_v_β_3_ on the tumor microvessels and the pattern of PTEN expression in these stage 3 neuroblastomas. Analysis showed significant difference between the mean percentage of microvessels expressing integrin α_v_β_3_ in tumors with diffuse PTEN compared to those with focal or negative PTEN expression (diffuse PTEN: mean 32% of microvessels expressed α_v_β_3_, 95% CI 23%–42%, *n* = 28 *vs*. focal or negative PTEN: mean 60% of microvessels expressed α_v_β_3_, 95% CI 51%–69%, *n* = 25; *p* < 0.001; Figure 2B and 2E). Thus, pattern of expression of PTEN differs between aggressive and less aggressive stage 3 neuroblastomas, such that aggressive stage 3 neuroblastomas are more likely to express α_v_β_3_ on the majority of their microvessels and only express limited PTEN on the tumor cells.

### PTEN regulates neuroblastoma growth in mice

**Figure 3:**
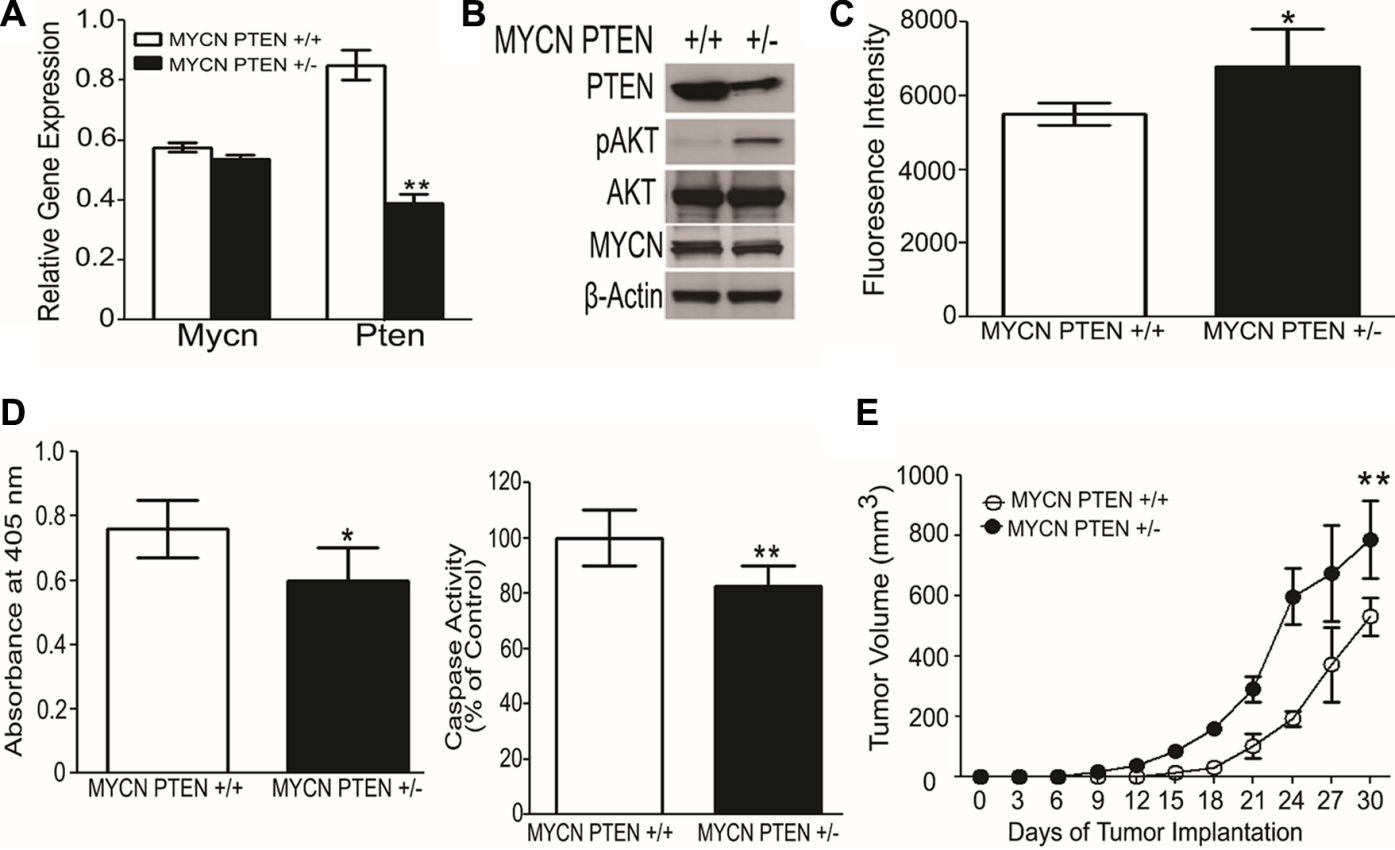
Loss of one allele of PTEN promotes neuroblastoma growth in mice (**A**) Quantitative RT-PCR shows lower Pten mRNA in neuroblastoma-derived cell lines obtained from MYCN PTEN+/− compared with MYCN PTEN+/+ mice. *Mycn* mRNA levels were similar between the two lines. Values reflect *Mycn* and *Pten* mRNA relative to *Gapdh*, analyzed in triplicate. (**B**) Western blot analysis showing the protein level of PTEN and MYCN in cell lines obtained from neuroblastomas in MYCN PTEN+/+ and MYCN PTEN+/− mice. (**C**) Cells from MYCN PTEN+/− mouse neuroblastomas show more rapid increase of viable cell number in culture compared to neuroblastoma cells from MYCN PTEN+/+ mice as analyzed by AlamarBlue^®^ described in Methods. (**D**) Left panel shows cell death ELISA assay performed on MYCN PTEN+/+ and MYCN PTEN+/− neuroblastoma cells according to manufacturer's protocol. Right panel shows caspase 3 activity done in triplicates in MYCN PTEN+/+ and MYCN PTEN+/− neuroblastoma cells. (**E**) 5 × 10^6^ tumor derived neuroblastoma cell lines obtained from MYCN PTEN+/+ and MYCN PTEN+/− mice were inoculated subcutaneously in nude mice (*n* = 7–8 mice per group). Graphs present mean ± SEM of 7–8 mice. Statistical significance is assessed by two sample *t*-test where *denotes *P* < 0.05, ** denotes *P* < 0.01 and *** denotes *P* < 0.001.

To examine a possible role for PTEN in neuroblastoma growth we mated MYCN transgenic mice, which spontaneously develop neuroblastoma tumors [41], with PTEN+/− mice, to achieve MYCN PTEN+/− *vs*. MYCN PTEN+/+ mice. The tumors were generated in the MYCN PTEN+/+ and MYCN PTEN+/− mice at different times. Moreover, the time of onset and location of spontaneous tumors in this mouse are impossible to predict, hence, it is logistically very difficult to test drugs in this spontaneous tumor model. For this reason, we established tumor cell lines from spontaneous murine MYCN Tg tumors which were PTEN +/+ vs PTEN +/−in order to examine genetics of PTEN haploinsufficiency and AKT activation on tumor growth in a syngeneic genetic model. Messenger RNA of cell lines derived from the spontaneously-arising neuroblastoma tumors confirmed reduced *Pten* mRNA in MYCN PTEN+/− cells compared to MYCN PTEN +/+ cells, without difference in *Mycn* mRNA levels (Figure 3A). Western blot similarly showed reduced expression of PTEN, as well as elevated levels of phosphorylated AKT (pAKT) in the MYCN PTEN+/− cells, and no difference in expression of MYCN (Figure 3B). When grown in culture, the MYCN PTEN+/− neuroblastoma cells consistently showed higher AlamarBlue fluorescence intensity compared to MYCN PTEN+/+ tumor cells, indicating a higher number of viable MYCN PTEN+/− cells (Figure 3C) and supporting a key inhibitory role for PTEN in growth of neuroblastoma cells *in vitro*. Consistent with this, cell death ELISA and caspase 3 assays both showed that MYCN PTEN+/−neuroblastoma cells underwent less apoptosis as compared with MYCN PTEN+/+ tumor cells (Figure 3D). Finally we tested if decrease in PTEN promoted neuroblastoma tumor growth *in vivo*. For this, MYCN PTEN+/+ and MYCN PTEN+/− neuroblastoma cells were implanted into the flank of nude mice and tumor growth was monitored for 30 days. Results establish that loss of one copy of PTEN promoted neuroblastoma tumor growth compared to tumors retaining both copies of PTEN (Figure 3E). These results suggest that PTEN has a growth-regulatory role in a MYCN-driven neuroblastoma model system.

### SF1126 has potent PI3K/BRD4 inhibitory activity in NB models

**Figure 4:**
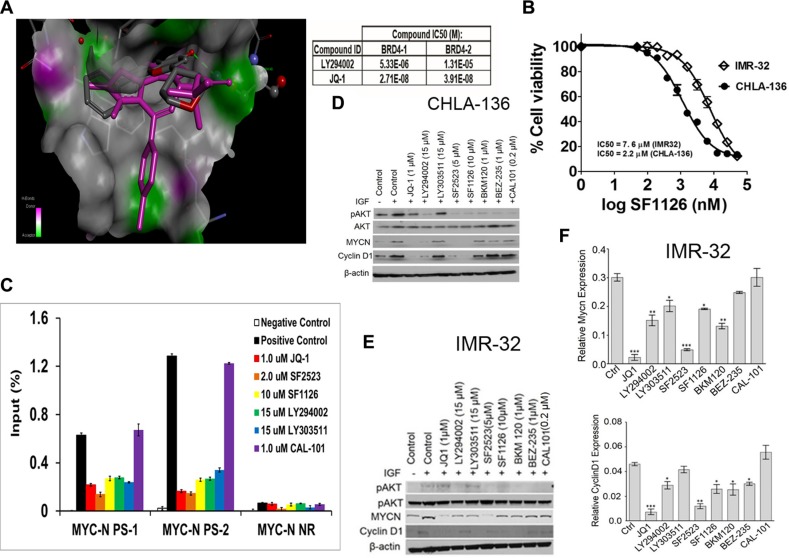
MYCN expression is directly regulated by BRD4 and repressed by SF1126 treatment (**A**) Left panel shows molecular modelling diagram depicting molecular interaction between LY294002, the active moiety of SF1126, and the BRD4 bromodomain binding domain 1 (BD1). Right panel shows alpha screen binding assay performed using Histone H4 peptide (1–21) K5/8/12/16Ac-Biotin as a ligand. (**B**) IC50 curves for IMR-32 or CHLA-136-Fluc cells treated with SF1126 using Alamar Blue as described in Methods. (**C**) ChIP with a BRD4 antibody at 2 sites within the *MYCN* promoter region in IMR-32 cells treated with 1 μM JQ1, 5 μM SF2523, 10 μM SF1126, 1 μM CAL-101 for 24 hours. Error bars are ± SEM from triplicate experiments. *P* < 0.05 as compared to positive control (paired *t*-test). Positive control: no inhibitor; IP with anti-BRD4 antibody, Negative control: no inhibitor, IP with rabbit anti-IgG antibody. (**D**–**E**) CHLA or IMR-32 cells were serum starved for 4 hrs followed by treatment with indicated inhibitors for 24 hrs. Cells were stimulated with 50 ng/ml IGF and used for lysate preparation for Western blot analysis after 24 hrs of treatment with various inhibitors. (**F**) Real Time PCR data showing effect of various inhibitors on gene expression of Mycn and Cyclin D1 as described in Methods. Data was normalized to GAPDH. Graphs represent mean ± SEM. Data was analyzed by student's *t*-test, where ***P < 0.001, **P < 0.01, *P < 0.05 vs. ctrl (DMSO).

We next asked if interference with PTEN signaling downstream of PI3K/AKT could interfere with growth of neuroblastoma xenografts. A previous report demonstrating that LY294002, the active moiety of SF1126, was a BRD4 inhibitor in a examine if BRD4 binding domain 1 BD1 binding assay prompted us to confirm that SF1126 inhibited BRD4 and in our *in vitro* and *in vivo* models. We used molecular modeling of the BRD4 binding domain 1 (BD1) crystal structure coordinates to examine the binding mode of LY294002 within the acetyl-lysine binding pocket as compared to another well characterized BRD4 inhibitor, JQ1 [25]. We created an *in silico* model of BRD4-BD1 with LY294002 and JQ1 (PDB code: 3MXF) to obtain their free binding energy (ΔG°, kcal/mol) and binding mode at the BRD4-BD1 active site (Figure 4A, Left panel). Our *in silico* docking results showed that LY294002 (BRD4-BD1 IC_50_ = 5.3 μM) and JQ1 (BRD4-BD1 IC_50_ = 33 nM) bound to BRD4-BD1 with an almost identical orientation and conformation as they are found in their corresponding BRD4-BD1 crystal structures. Similarly, the trend of their predicted binding affinity (binding scores = −14.808 and −24.956 kcal/mol, respectively) is in accordance to their BRD4-BD1 inhibitory potency *in vitro* binding assays. The alpha screen binding assay using BD1 domain of BRD4 performed in collaboration with Reaction Biology demonstrated BRD4 inhibitory activity of LY294002 and JQ1 using Histone H4 peptide (1−21) K5/8/12/16Ac-Biotin as a ligand (Figure 4A, Right panel) of 5 μM and 33 nM, respectively for BD1. We next investigated effect of SF1126 on MYCN amplified neuroblastoma cell lines IMR-32 and CHLA-136. These cell line responded to SF1126, which conferred a dose-responsive, inhibitory effect on cell viability (Figure 4B). The IC_50_ for IMR-32 and CHLA-136 was found to be 7.6 μM and 2.2 μM respectively. It was previously shown that the BET bromodomain inhibitor, JQ1 displaces BRD4 from the MYCN promoter region so we investigated if SF1126 is able to displace BRD4 from MYCN promoter. Using chromatin immunoprecipitation (ChIP) PCR, we observed BRD4 localization to the transcriptional start site of MYCN in IMR-32 cells, as well as a putative enhancer region. Similar to JQ1, SF1126 treatment resulted in displacement of the BRD4 co-activator protein from both elements, providing a mechanistic explanation for the observed SF1126-dependent decrease in MYCN transcription in IMR-32 cells (Figure 4C). In order to provide the specificity of bromodomain inhibitor in blocking the expression of MYCN, we used a panel of inhibitors viz. BEZ-235 [42] (Selleck chemicals), and BKM120 [43] (Novartis), Cal101, LY294002, SF1126 [22] (SignalRx), SF2523 [44] (SignalRx), JQ1 (Selleck Chemicals) and LY303511 [45]. Among these, BEZ-235, BKM120, Cal101 are known PI-3K inhibitors. Importantly, they display no BRD4 inhibitory activity (unpublished data). In contrast, LY294002, SF1126, SF2523 inhibit both PI-3K and BRD4, whereas JQ1 and LY303511 [45] are BRD4 inhibitors which do not inhibit PI-3K activity. It is recently reported that commonly used PI-3K inhibitor LY294002 is an inhibitor of BET bromodomains [25]. Results in Figure 4C depicts that PI-3K inhibitor Cal101 is unable to displace BRD4 from MYCN promoter region. Moreover, SF1126 treatment resulted in down regulation of MYCN in IMR-32 and CHLA-136 cells as revealed by Western blotting (Figure 4D and 4E) and RT-PCR (Figure 4F). As shown in Figure 4D and 4E, PI-3K inhibitors only blocked phosphorylation of AKT, JQ1 treatment only effect was on MYCN and Cyclin D1 levels without affecting p-AKT levels while LY294002, SF1126 and SF2523 affect both p-AKT and MYCN and its target expression suggesting greater potency of SF1126 in MYCN amplified tumors. Some recent evidence suggests that not just the MYCN is a target for BRD4 but a number of MYCN target genes are inhibited by BRD4 inhibitors, resulting in greater potency of JQ1 [46]. It is important to mention that the PI-3K inhibitors BEZ-235, BKM120, Cal101 showed no or very mild effect on MYCN and Cyclin D1 protein levels in CHLA-136 cells (Figure 4D), and IMR-32 cells (Figure 4E). The observed reduction in MYCN protein levels upon treatment with PI-3K inhibitors is due to the fact that PI-3K inhibition destabilizes MYCN protein [47].

### PI3K blockade inhibits growth of established neuroblastoma tumors *in vivo*

**Figure 5:**
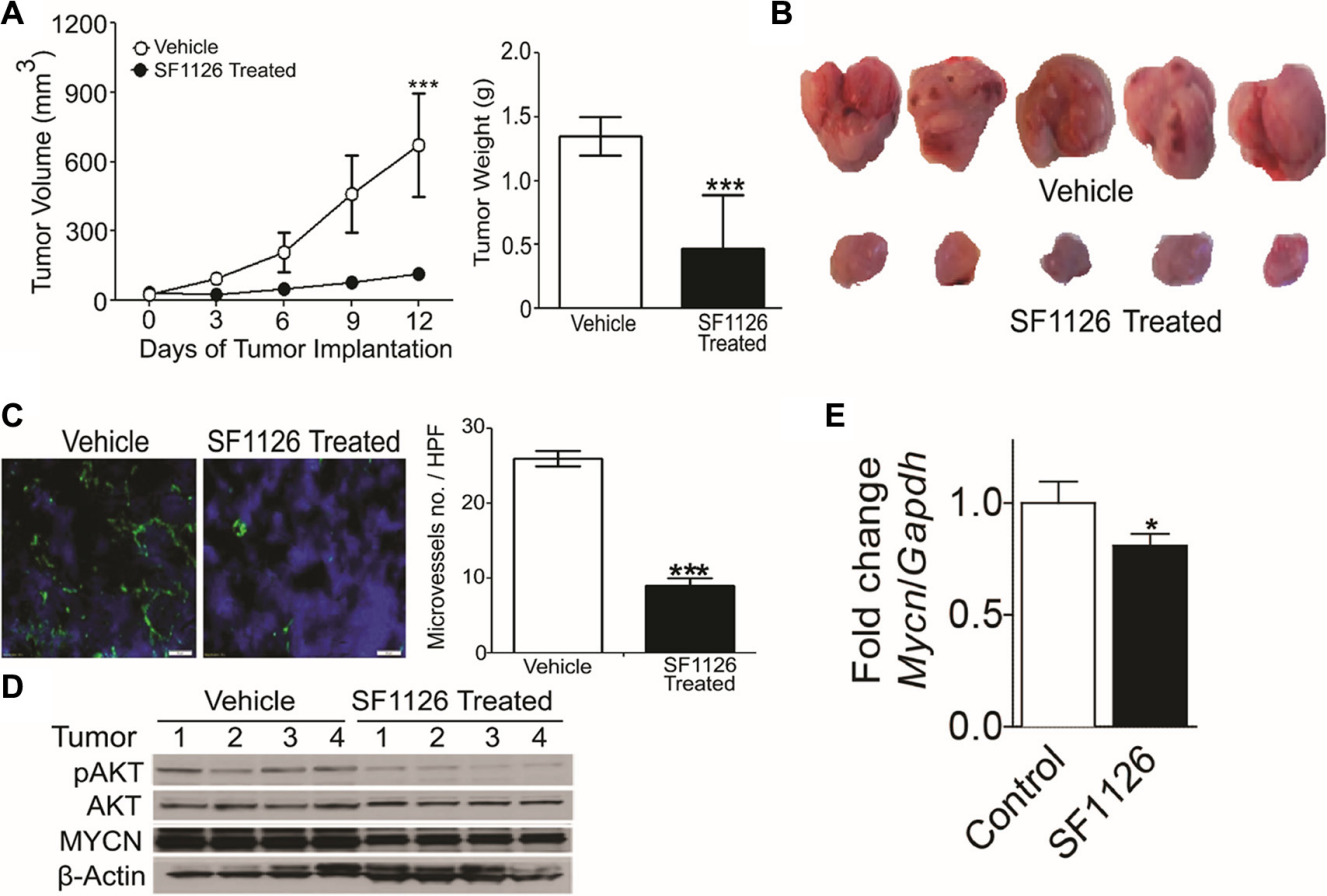
The RGD-targeted PI3K/BRD4 inhibitor SF1126 inhibits growth and microvessel density in neuroblastoma xenografts while decreasing AKT phosphorylation and MYCN protein (**A**) Subcutaneous NB9464 tumors in *nu/nu* mice (*n* = 7–8 mice per group) were treated with 50 mg/kg/dose SF1126 or vehicle SQ x5/week starting 24 days after tumor inoculation, until tumors were harvested. Left panel show sequential tumor volumes and right panel shows the weights of the tumors harvested on day 30. Values are mean ± SEM (*p* < 0.001; pair wise two-sided Student's *t* test). (**B**) Representative images of the tumors isolated from the mice in A. (**C**) Left panel shows representative CD31 (green) immunofluorescence staining of tumor vasculature with counterstain by DAPI (blue) using frozen sections of NB9464 tumors from panel A–B. Right panel shows reduced microvascular density (MVD) in tumors from SF1126- compared to vehicle-treated mice. *P < 0.001 vs. vehicle treated animals. (**D**) Western blot analysis of MYCN and pAKT on tumors isolated from the SF1126- or vehicle-treated mice in A–B. (**E**) Quantitative RT-PCR of *Mycn* mRNA relative to *Gapdh* in tumors isolated from mice in A treated with SF1126 or with vehicle, normalized to the control tumors. Shown are mean fold change ± SEM, *n* = 4 per group, *p* = 0.013.

**Figure 6:**
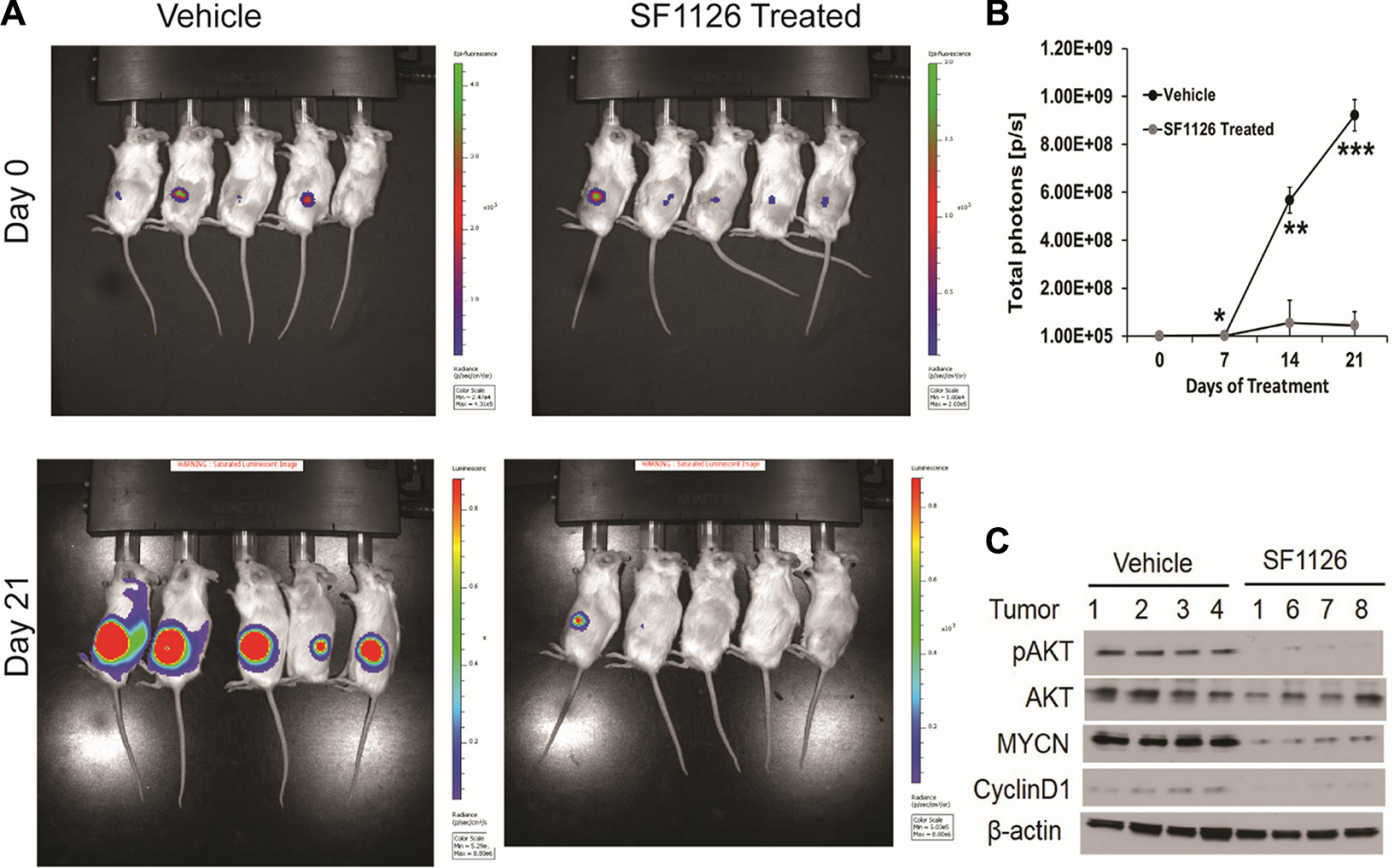
SF1126 inhibits tumor growth of MYCN amplified CHLA-136 xenografts *in vivo* (**A**) 5 × 10^6^ CHLA-136-Fluc cells implanted subcutaneously in NSG mice. After 15 days of tumor implantation, mice were imaged on Xenogen IVIS-200 system & mice were randomized into vehicle & SF1126 treated groups. Mice were treated with SF1126 (50 mg/kg/day five days a week) for 3 weeks, until tumors were harvested. Figure shows the longitudinal images of a representative mouse from each group visualized on day 0 and day 21 (of SF1126 treatment started) using bioluminescence imaging. (**B**) Quantitative analysis of bioluminescence signals in Vehicle and SF1126 treated group using IVIS Spectrum. (**C**) Western blot analysis of pAKT, MYCN and Cyclin D1 on tumors isolated from the SF1126- or Vehicle-treated mice in A.

Above results demonstrate that 1) integrin α_v_β_3_ expression on microvessels in stage 3 neuroblastoma is increased in the more aggressive tumors and is associated with focal or negative PTEN expression in these tumors, 2) SF1126, has potent PI3K/BRD4 inhibitory activity suggesting that this pathway may be an effective therapeutic target in neuroblastomas. These finding prompted us to examine the effect of the dual PI3K/BRD4 inhibitor SF1126 on neuroblastoma tumor growth *in vivo*. For this, we used NB9464 and CHLA-136 neuroblastoma cells. We injected NB9464 murine neuroblastoma cells into flanks of nude mice and when tumors grew to approximately 40 mm3 we treated them with SF1126 or vehicle five times a week until criteria for euthanasia were reached. In mice treated with SF1126 tumor growth was significantly decreased in comparison with vehicle controls (Figure 5A–5B) (*p*-value 0.006). The reports that high MYCN is associated with enhanced tumor angiogenesis and poor clinical outcome in neuroblastoma [3] and the known antiangiogenic activity of SF1126 [22] prompted us to explore a possible effect of SF1126 on the microvasculature of these NB9464 neuroblastomas. CD31 staining indeed, showed that microvessel density was significantly reduced in tumors from mice treated with SF1126 compared to vehicle (Figure 5C). Phosphorylation of AKT (p-AKT) was lower in the SF1126-treated tumors compared to vehicle controls, suggesting that SF1126 indeed inhibited its molecular target *in vivo*. Lastly, MYCN protein and mRNA were also lower in the SF1126-treated tumors (Figure 5D–5E). In a separate set of experiments, CHLA-136 was injected in NSG mice and after 15 days of tumor inoculation, when all mice showed tumor growth (Figure 6A), mice were randomly separated into two groups and were treated with 50 mg/kg of SF1126 (5 times a week) for 3 weeks. The day when treatment began was considered as day 0. Figure 6A clearly depicts that on day 21 of treatment tumors were completely regressed in SF1126 treated group as compared to control. Figure 6B shows the significant reduction in bioluminescence signal in SF1126 treated group inoculated with CHLA-136-Fluc tumor cells compared to control on 7, 14 and 21 day of SF1126 treatment. Western blot analysis done on Vehicle and SF1126 treated group tumors showed reduced protein levels of pAKT, MYCN and Cyclin D1 in treated tumors (Figure 6C). These data show that SF1126 blocked PI-3K signaling, decreased MYCN, and diminished angiogenesis in the tumors, suggesting that SF1126 may provide benefit in treatment of aggressive neuroblastomas.

## DISCUSSION

In previous work we showed that stage 4 neuroblastoma tumors express the angiogenic integrin, α_v_β_3_ on their endothelial cells in a higher proportion of their microvessels compared to stage 1 and 2 tumors [5]. The small cohort of stage 3 neuroblastomas in our prior analysis showed α_v_β_3_ expression on 87% of microvessels in the MYCN-amplified neuroblastomas (high risk group; *n* = 3) but only on 20% of microvessels in the non-MYCN amplified tumors (intermediate risk group; *n* = 2), suggesting possible correlation between microvascular α_v_β_3_ expression and risk group in the stage 3 neuroblastoma tumors. Our results here expand these findings to a group of 54 stage 3 neuroblastomas, and show that 68% (mean) of microvessels in the aggressive stage 3 tumors (MYCN amplified/unfavorable histology) expressed integrin α_v_β_3_ compared to only 34% (mean) in the non-MYCN amplified/favorable histology ones. These data provide the first evidence for a difference in angiogenic characteristics within the more aggressive *vs*. less aggressive stage 3 neuroblastomas. These data further suggest that angiogenesis plays a critical role in the biology of high-risk neuroblastomas [3, 48] and that integrin α_v_β_3_ and its upstream and downstream signaling effectors may be both biomarkers and potential targets for therapeutic consideration [5].

In our current and prior studies, as well as in tumor types studied by others, integrin α_v_β_3_ was expressed on the angiogenic endothelial cells and was associated with a more malignant tumor phenotype [5, 49, 50]. In our current series of stage 3 neuroblastomas the expression of integrin α_v_β_3_ on tumor microvessels was predictive of survival in a univariate analysis, but not after adjusting for MYCN and Shimada classification. This suggests that angiogenesis (and endothelial integrin α_v_β_3_ expression) may be linked to the same biological pathway(s) by which MYCN and Shimada classification are linked to tumor behavior and prognosis. Perhaps a more important conclusion from this study would be that MYCN, α_v_β_3_ and PTEN are important predictive biomarkers to be used in the application of dual PI-3K/BRD4 inhibitors for this disease subgroup within high risk NB. This is the case in our currently ongoing Phase I study of SF1126 in recurrent NB.

In this series of intermediate-risk stage 3 neuroblastomas, we showed that PTEN, a tumor suppressor and critical upstream regulator of the PI3K/AKT pro-survival pathway, was diffusely expressed on tumor cells. Consistent with our finding, AKT activation is thought to be a negative prognostic indicator in neuroblastoma [51]. Interestingly, dominant negative AKT as well as PTEN are negative regulators of integrin α_v_β_3_ function in cultured endothelial cells [37] and PTEN has anti-angiogenic function [16, 17]. Moreover, the inhibitory effect of a fragment of tumstatin (a cleavage product of collagen IV that is an endogenous angiogenesis inhibitor) on integrin α_v_β_3_ was highly dependent on expression PTEN and regulation of the AKT pathway [18]. This is consistent with our current data, that in tumors with diffuse expression of PTEN, fewer microvessel expressed the angiogenic integrin α_v_β_3_ and biologically these low-α_v_β_3_ stage 3 neuroblastomas were mostly intermediate-risk rather then high-risk ones. Our findings in the neuroblastomas of MYCN PTEN+/− mice further validate the role of PTEN in the potential pathogenesis of human neuroblastoma (Figure 3).

The present study shows that microvascular expression of α_v_β_3_ integrin is correlated with decreased expression of the tumor suppressor *PTEN* in stage 3 neuroblastoma tumors (Figure 1). Moritaki *et al*. reported that *PTEN* was mutated in the KP-N-AYR neuroblastoma cell line, which was established from bone marrow metastases of a 2½ year old patient with stage 4 neuroblastoma at time of recurrence following chemotherapy. Interestingly, the original KPN-AY cell line, established at diagnosis from this patient, had intact *PTEN* [52, 53]. Comparative microarray studies in these isogenic KPN-AY and KPN-AYR cell lines in our laboratory confirmed loss of *PTEN* expression in K-PN-AYR cells and dramatic alterations in its transcriptome (unpublished data) and drug sensitivity [53] compared to K-PN-AY. Figure 2 showed that loss of one allele of PTEN enhanced tumorigenecity of MYCN-driven neuroblastomas. Figures 5 and 6 showed that administration of SF1126 in the neuroblastoma tumor models decreased angiogenesis and tumor growth and decreased both MYCN mRNA and protein. These findings are in line with reports that inhibition of PI3K along with destabilization of MYCN block malignant tumor progression in neuroblastoma [47, 54, 55]. These findings led us to hypothesize that administration of an integrin-targeted PI3K inhibitor may provide an effective strategy to treat neuroblastoma patients while avoiding some of the side effects of systemically-administered PI3K inhibitors. SF1126, a pan PI3K inhibitor that is an RGDS-conjugated LY294002 prodrug engineered to bind to the αvβ3 integrin, has shown anti-tumor and anti-angiogenic activity in a number of xenograft models [19–23]. In the present study we found that SF1126 displaces BRD4 from MYC transcriptional start site in IMR-32 neuroblastoma cell line (Figure 4C). Molecular modelling and BRD4 binding studies demonstrate LY294002 binding to the BRD4 BD1 bromodomain. These findings suggest a novel and interesting function of this drug as it can be used to target multiple signaling nodes at the same time. Moreover, this drug has increased solubility and binds to specific integrins (α_v_β_3_) within the tumor compartment, resulting in enhanced delivery of the active compound to the tumor and its vasculature [22]. SF1126 has recently shown considerable efficacy in adult solid tumors and B cell malignancies in Phase I clinical trials, and is entering phase II trials in adults [24]. In 2015 SF1126 entered pediatric Phase I clinical trials for neuroblastoma via the NANT (New Approaches to Neuroblastoma Therapy) consortium, and represents the first PI3K inhibitor to enter pediatric oncology clinical trials.

In summary, our data show that aggressive and less aggressive stage 3 neuroblastomas differ in terms of microvessel expression of the angiogenic integrin α_v_β_3_, and that the decreased expression of the tumor suppressor PTEN is associated with increase in microvascular integrin α_v_β_3_ expression. Finally we showed that the integrin-targeted dual pan- PI3K/BRD4 inhibitor, SF1126, potently blocked tumor growth and tumor angiogenesis along with decreasing MYCN mRNA and protein in subcutaneous neuroblastoma xenografts. These findings suggest that metronomic antiangiogenic therapy with inhibitors of PI3K, as part of multi-modality therapy, may be useful against high-risk neuroblastoma and that MYCN, α_v_β_3_, PTEN and p-AKT will represent potential biomarkers to use in the design of ongoing Phase I/II trials of SF1126 in neuroblastoma therapeutics.

## MATERIALS AND METHODS

### Cells, reagents and antibodies

IMR-32 cells were obtained from ATCC and maintained in DMEM/10%FBS. CHLA-136--Fluc neuroblastoma cells were provided by Dr. Robert Seeger (Children's Hospital Los Angeles) and were maintained in RPMI-1640/10% FBS [56]. The CHLA-136 cell line is a particularly chemoresistant cell line with MYCN amplification and was established from the peripheral blood of a patient after chemotherapy and bone marrow transplantation [56]. The NB9464 disialoganglioside-2-positive, MYCN-overexpressing murine neuroblastoma cell line, maintained in RPMI-1640/10%FBS, was a kind gift from Dr. Jon Wigginton (NCI), in whose laboratory it was derived from spontaneous neuroblastoma tumors arising in C57BL/6 MYCN transgenic mice developed originally by Dr. William A. Weiss (University of California, San Francisco, CA) [57]. All cell lines used in the study were authenticated by short tandem repeat DNA profiling at the respective cell banks and were maintained as recommended by the suppliers. The MYCN-PTEN+/+ and MYCN-PTEN+/− murine neuroblastoma cell lines were isolated from spontaneously-arising neuroblastomas in MYCN-PTEN+/+ and MYCN-PTEN+/− mice, respectively, as described below, and used up to passage 5. Drugs or vehicle controls were added after cell spreading, 2−6 h after seeding. Reagents were purchased from Sigma Chemical Company (St. Louis, MO) unless stated otherwise. Affinity purified monoclonal antibody to integrin αvβ3 (LM609) was a generous gift from Dr. David Cheresh [58]. Monoclonal anti human CD31 (1A10, catalog# CMC338) was from Cell Marque, (Rocklin, California), mouse CD31 was from BD Biosciences, Alexa488 was from Invitrogen, PTEN (sc-7974; clone A2B1) was from Santa Cruz Biotechnology Inc. (Santa Cruz, CA) and isotype-specific mouse IgG1 (control) was from Dako Corporation (Carpinteria, CA). Secondary antibody for immunohistochemistry was multilink (swine) anti-goat, -mouse, -rabbit immunoglobulins (catalog# E0453) from Dako Corporation (Carpinteria, CA). Avidin-biotin-peroxidase (catalog# PK400) was from Vector Laboratories Inc. (Burlingame, CA).

### Cell growth/cell numbers and apoptosis studies

4 × 10^4^ IMR-32 and CHLA-136 cells were grown in 96 well plate for overnight, then treated with SF1126 (0.0978 μM − 100 μM) for 48 hrs followed by addition of Alamar Blue and incubation of plate at 37°C in 5% CO_2_ incubator for 6 hrs. Fluorescence signals were read as emission at 590 nm after excitation at 560 nm as described before [59]. Proportion of viable MYCN PTEN+/+ and MYCN PTEN+/− neuroblastoma tumor cells were assessed by plating cells at 1 × 10^4^ cells/well in 96-well plates. Cells were incubated for 48 hrs followed by addition of AlamarBlue^®^ (Roche) and reading of fluorescence signals as described above.

Cell death ELISA detection (Roche Applied Science) was used to quantify histone-complexed DNA fragments (nucleosomes) in cytoplasm of the apoptotic cells after induction of apoptosis as described before [59]. Briefly, 1×10^4^ MYCN PTEN +/+ and MYCN PTEN +/− cells were seeded in 96 well plate. After 24 hrs, cells were processed for Cell death detection ELISA assay followed manufacturer's protocol (Roche Diagnostics GmbH, Mannheim, Germany). Absorbance was measured at 405 nm wavelength using a fluorescence spectrophotometry (Infinite M200, Tecan Instruments, Germany).

For caspase-3 activity (apoptosis studies), MYCN PTEN +/+ and MYCN PTEN+/− (2 × 10^6^) cells were seeded in 10 cm dish. After 24 hours cells were collected, and caspase-3 activity was assessed with the Caspase-3 assay kit following manufacturer's protocol (Roche). Fluorescence intensity was measured by fluorescence spectrophotometry (Infinite M200, Tecan Instruments, Germany) at 400 nm excitation and 505 nm emission wavelengths. All readings were standardized using the fluorescence intensity of an equal volume of free 7-amino-4- methyl-coumarin (AMC) solution.

### DNA Methylation

DNA methylation was assessed using the MethyLight method as described [60] using a reaction that was specific for the PTEN gene promoter, and avoided the pseudogene [61]. MethyLight reactions for RASSF1A, known to be methylated in neuroblastomas [39], CDKN2A and MTHFR were used as methylation controls. MethyLight reactions for the ACTB and COL2A1 genes were used as loading controls. Human genomic DNA that was artificially methylated using M.*Sss*I CpG methyltrase served as a methylated reference.

### Generation of MYCN-PTEN+/− neuroblastoma cell lines

In order to study the role of PTEN in neuroblastoma tumorigenesis, MYCN transgenic mice [41] were crossed with PTEN+/− mice (gift from Ramon Parsons, Columbia University, New York, NY) [62]. MYCN transgenic mice were obtained from William Weiss, University of California, San Fransisco. The generation of the MYCN transgenic mouse model, which shows spontaneous emergence of neuroblastoma tumors and is based on targeted expression of the Mycn oncogene to mouse neuroectodermal cells via the mouse tyrosine hydroxylase promoter, has been described previously [41]. For simplicity, MYCN mice will be referred to as MYCN-PTEN+/+ and ones crossed with PTEN+/− mice will be referred as MYCN-PTEN+/−mice. Spontaneous palpable neuroblastoma tumors obtained in MYCN-PTEN+/+ and MYCN-PTEN+/− transgenic mice were dissociated and processed for stable cell line preparation. Briefly, tumor tissue was cut into small pieces, and incubated at 37°C for 30 min in digestion buffer consisting of Dulbecco's PBS (DPBS, Life Technologies, Grand Island, NY) with 10 U/ml papain (Worthington, Lakewood, NJ), 200 μg/ml L-cysteine, and 250 U/ml DNase (Sigma, St. Louis, MO). The digestion buffer was then removed and replaced with DPBS containing 8 mg/ml soybean trypsin inhibitor (Boehringer Mannheim, Indianapolis, IN), 8 mg/ml bovine serum albumin (BSA, Sigma), and 250 U/ml DNase, followed by trituration of tissue using pipettes of decreasing bore size to obtain a single-cell suspension. Cells were centrifuged at room temperature and resuspended in PBS containing 200 μg/ml BSA (PBS/BSA) and passed through a cell strainer (Becton Dickinson, Franklin Lakes, NJ) to remove debris. This suspension was centrifuged through a step gradient of 35% and 65% Percol (Amersham Biosciences), and cells were harvested from the 35%–65% interface, washed in PBS/BSA and allowed to grow in media containing DMEM + 10% FBS + M3 base (INCELL M300 A-500). These tumor derived neuroblastoma cell lines were passaged and used for *in vivo* studies at passage 5 after isolation from the tumors.

### Immunohistochemistry and Immunofluorescence

Preparation of serial cryostat sections (6 μm) and staining were as described [5]. Primary antibody concentrations were LM609 anti- αvβ3 1:500 (4), PTEN 1:50, and Anti-CD31 1:50 (2 h). Secondary antibody was used at 1:50. Mouse IgG1, used as a negative control, was negative in all cases. For immunofluorescence studies, cryosections were incubated with primary antibodies against CD31, followed by Alexa488 (green) labeled secondary antibody. The sections were counter-stained with DAPI to visualize nuclei and micro vascular density was measured in 40X fields photographed using Metamorph image capture and analysis software (version 6.3 r5, Molecular Devices). For CD31 staining microvessel density (MVD) was determined by counting the number of microvessels per high-power field (HPF) in the section with antibody reactive to CD31 as described before [63]. Microvessels were counted blindly in 5–10 randomly chosen fields.

### Quantification of gene expression

Total RNA was isolated from NB9464 tumors or IMR-32 cells treated with different conentrations of various inhibitors for 24 hrs using the Qiagen RNAeasy kit (Qiagen, Hilden, Germany) according to manufacturer's instructions. cDNA was prepared from 1 μg RNA sample using iscript cDNA synthesis kit (Bio-Rad, Hercules, CA). cDNA (2 μL) was amplified by RT-PCR reactions with 1× SYBR green supermix (Bio-Rad, Hercules, CA) in 96-well plates on an CFX96 Real time system (Bio-Rad, Hercules, CA), using the program: 5 min at 95°C, and then 40 cycles of 20 s at 95°C, 1 min at 58°C and 30 sec at 72°C. Specificity of the produced amplification product was confirmed by examination of dissociation reaction plots. Relative expression levels were normalized to *Gapdh* expression according to the formula < 2^(Ct gene of interest-Ct *Gapdh*) > [64].

### Western blotting

For all Western blots, 2 × 10^6^ IMR-32 or CHLA-136 cells were plated in 10 cm tissue culture dishes and were allowed to adhere for overnight. The cells were then serum starved for 4 hrs, stimulated with 50 ng/ml IGF and used for lysate preparation after 30 minutes of treatment with JQ1 (1 μM), LY294002 (15 μM), LY303511 (15 μM), SF2523 (5 μM), SF1126 (10 μM), BKM120 (1 μM), BEZ235 (1 μM) or CAL101 (200 nM). Whole cell lysates were prepared using RIPA buffer containing protease inhibitor cocktail (Roche Molecular Biochemicals). Clarified lysates were resolved in 10% SDS-PAGE, transferred to PVDF membrane and probed for different antibodies for p-AKT (Ser473), AKT, MYCN, Cyclin D1 and β-actin antibodies.

### Molecular modelling of SF1126/LY294002 in BRD4 BD1 site and BRD4 binding assays

The crystallographic atomic coordinates of BRD4-BD1 co-crystallized with JQ1 (PDB code 3MXF) were obtained from the Protein Data Bank [65]. To model the binding of LY294002 and JQ1 at the key acetyl-lysine recognition pocket, the PDB file was imported into LeadIT [BioSolveIT GmbH, An der Ziegelei 79, 53757 Sankt Augustin, Germany], all water molecules were kept, residues around JQ1 within a grid of 7 Å^3^ were selected and used for *in silico* docking calculationsThe 3D structures of LY294002 and JQ1 (all hydrogens included) were docked using LeadIT's standard parameters Compounds LY294002 and JQ1 were tested for BRD4-1 and BRD4-2 activity by using Histone H4 peptide (1-21) K5/8/12/16Ac-Biotin as a ligand in alpha screen binding assay. The test was performed in collaboration with Reaction Biology.

### CHIP analysis

IMR-32 cells were treated with/without JQ1 (1 μM), SF2523 (2 μM), SF1126 (10 μM), LY294002 (15 μM), LY303511 (15 μM) and CAL 101 (1 μM) for 24 hours and then cross-linked using 1.1% formaldehyde, washed with PBS and frozen at −80°C. Antibody-conjugated beads were prepared by blocking 50 μL of protein A/G agarose beads with 0.5% BSA (w/v) followed by incubation with 6.25 μg of anti-BRD4 antibody, 5 μg of normal rabbit IgG. Cross-linked cells were lysed, washed and sonicated, essentially as described [29, 33, 66]. Sonicated lysates were supplemented with Triton X-100 to 1% and cleared. Aliquots were reverse-crosslinked and digested with RNase A overnight and purified with QIAquick PCR Purification Kit (Qiagen, Hilden, Germany) for quantification of input chromatin. Sonicated, cleared chromatin (15 μg) was incubated overnight at 4°C with antibody-conjugated agarose beads, and beads were washed as in [29, 33]. Chromatin was eluted in the buffer (50 mM Tris-HCl pH 8, 10 mM EDTA, and 1% SDS), reverse cross-linked and digested with RNase A overnight and then purified. ChIP and input DNA were analyzed by real-time PCR analysis using previously published primers against the *MYCN* promoter site 1 (forward) TTTGCACCTTCGGACTACCC and (reverse) TTTGACTGCGTGTTGTGCAG; *MYCN* promoter site 2 (forward) TCCTGGGAACTGTGTTGGAG and (reverse) TCCTCGGATGGCTACAGTCT; *MYCN*-negative region (forward) TATCACCGTCCATTCCCCG and (reverse) TTGGAGGCAGCTCAAAGACC [29, 33]. Fold enrichment was analyzed by calculating the immunoprecipitated DNA percentage of input DNA in triplicate for each sample.

### Mice and *in vivo* studies

Mouse experiments were performed in accordance with animal protocols approved by the Institutional Animal Care and Use Committee at University of California, San Diego. To study the role of PTEN in neuroblastoma tumor progression, 5 × 10^6^ neuroblastoma-derived tumor cells obtained from MYCN PTEN+/+ or MYCN PTEN+/−transgenic mice were implanted subcutaneously in 6 week old female *nu*/*nu* mice. Tumor growth was monitored 2−3 times a week, until tumors were harvested on day 30. Tumor volume was calculated as: Volume = 0.5 × length × (width)^2^. For SF1126 experiments, 5 × 106 NB9464 murine neuroblastoma cells were injected subcutaneously in female *nu*/*nu* mice. When tumors reached 40 mm^3^, animals were randomized to two groups and SF1126 (50 mg/kg) or vehicle was administered subcutaneously five times a week. For CHLA-136-Fluc experiments, 5 × 10^6^ cells were subcutaneously implanted in NSG mice and after 15 days of tumor implantation, mice were randomized into two groups and one group is vehicle and another group is treated with 50 mg/kg SF1126 (five times a week) for 3 weeks. Tumor growth was assessed weekly by bioluminescence imaging 15 minutes after intra-peritoneal injection of a D-luciferin potassium salt solution (1.5 mg/mouse) using a Xenogen IVIS-200 system (Caliper Life Sciences). Photons emitted were quantified with the Living Image 3.0 software (Caliper Life Sciences).

### Patients and tumor specimens

Investigation was conducted in accordance with the ethical standards and according to the Declaration of Helsinki and national and international guidelines and was approved by the Institutional Review Board. Neuroblastoma specimens included in this study were resected at institutions of the Children's Cancer Group (CCG) between 1986–1996 under IRB-approved CCG protocols following informed consents and follow-up data was provided up to October 1997. Clinical staging was performed according to standard criteria used by the CCG at that time [14, 15]. Neuroblastoma tumor tissue processing was performed as described [5]. A total of 75 stage 3 anonymized neuroblastoma tumors were analyzed, including the five stage 3 tumors that we described previously [5]. Twenty one of the 75 samples processed were excluded either due to poor tissue preservation, extensive necrosis, or the tissue source not being the pre-therapy primary tumor at the time of diagnosis, leaving 54 evaluable tumors. One additional sample was not available for PTEN analysis. Patient and tumor characteristics for the evaluable 54 samples (53 for PTEN) are summarized in Table 1. DNA for methylation studies was available for 19 of the samples. Human neuroblastoma tumor gene expression analysis was performed with the help of R2: Genomics Analysis and Visualization Platform (http://r2.amc.nl; Academic Medical Centre, Amsterdam). Gene expression from 2 different cohorts of neuroblastoma patient samples quantified by microarray analysis were used with dataset GEO IDs: GSE49710 (498 samples) and GSE73517 (105 samples). Cutoff for PTEN low and high expression on Kaplan-Meier plots were automatically calculated by the scan modus.

### Statistical analyses and evaluation of immunohistochemistry

All laboratory and histopathological analyses were performed independently and without knowledge of clinical data. Immunohistochemical slides from sequential sections were analyzed by two observers who visually determined the proportion of microvessels in the whole section in which the microvascular endothelium specifically stained with the LM609 antibody (mAb), as compared to the total CD31-positive vessels in the contiguous section, as described [5]. Each observer repeated their analysis in a second, temporally-separated session, and was blinded to their previous reading, to the reading of the other observer, and to the clinical characteristics of the patients. Final score of α_v_β_3_ expression was the average between the values assigned by the two observers. The difference between the final scores assigned by the two observers in the 54 tumors was ≤ 25% in 45 tumors and > 25% in 9 tumors. PTEN was assigned either “diffuse”, “focal”, or “negative” values based on continuity and uniformity of the staining in the sample. Two-sample *t*-test was used to test whether expression of integrin α_v_β_3_, as measured by the percent of microvessels which stained with LM609 antibody, was associated with age, MYCN, Shimada classification, and PTEN expression pattern. Analysis of variance was used to compare expression of integrin α_v_β_3_ among the three risk groups defined by MYCN and Shimada classification (MYCN amplified and unfavorable Shimada, MYCN-non-amplified and unfavorable Shimada, or MYCN-non-amplified and favorable Shimada). Pair-wise comparisons between the risk groups were performed using the least significant difference method once the overall F-test was significant at α = 0.05. The associations between PTEN expression pattern and other prognostic factors were tested using Pearson Chi-square test. The log-rank test was performed to test the univariate associations of overall survival with expression of integrin α_v_β_3_, PTEN expression pattern, MYCN etc. The stratified log-rank test using the risk groups defined by MYCN and Shimada classification as the stratifying factor was also performed to examine if expression of integrin α_v_β_3_ and PTEN expression pattern were associated with overall survival, independently from MYCN and Shimada classification. Tissue culture experiments were performed at least three times unless stated otherwise and values represent means ± SEM. *P*-values were calculated by student's *t*-test (or where stated, one-way ANOVA) using GraphPad Prism version 4.0c for Mac (GraphPad Software, San Diego California USA, www.graphpad.com).

## Abbreviations

PTEN: Phosphatase and tensin homolog
PI3K: phosphoinositol-3 kinase
mTOR: mammalian target of rapamycin
mTORC: mTOR complex
